# Histone methyltransferase and drug resistance in cancers

**DOI:** 10.1186/s13046-020-01682-z

**Published:** 2020-08-28

**Authors:** Cheng Yang, Jiayu Zhang, Yukui Ma, Chunfu Wu, Wei Cui, Lihui Wang

**Affiliations:** 1 Department of Pharmacology, Shenyang Pharmaceutical University, Shenyang, People’s Republic of China; 2 Benxi Institute of Pharmaceutical Research, Shenyang Pharmaceutical University, Benxi, People’s Republic of China; 3 Shandong Academy of Pharmaceutical Sciences, Jinan, China

**Keywords:** Drug resistance, Cancer, Histone methyltransferase, Therapeutic strategy

## Abstract

A number of novel anticancer drugs have been developed in recent years. However, the mortality of cancer patients remains high because of the emergence of drug resistance. It was reported that drug resistance might involved in changes in gene expression without changing genotypes, which is similar to epigenetic modification. Some studies indicated that targeting histone methyltransferase can reverse drug resistance. Hence, the use of histone methyltransferase inhibitors or histone demethylase inhibitors opens new therapeutic approaches for cancer treatment. While the relationship between histone methyltransferase and tumor resistance has been determined, there is a lack of updated review on the association between them. In this review, we summarized the mechanisms of histone methyltransferases in cancer drug resistance and the therapeutic strategies of targeting histone methyltransferase to reverse drug resistance.

## Background

Epigenetics is a branch of biology and its concept was proposed by a British biologist named Conrad Waddington in 1939 [1, 2]. “Epigenetics” is refered to the inheritable changes of gene expression without alteration in DNA sequence [3]. With the expansion of the field of epigenetics, epigenetic modifications is divided into DNA methylation, histone methylation and acetylation according to the location and type of modifications [4]. It has been suggested that epigenetic modification is closely associated with cancer development [5, 6]. Histone methylation, which can be reversibly modified, has received a lot of attention. Histone methylation depends on histone methyltransferases (HMTs) in cells, which can reversibly methylate specific residues of histones, thereby regulating gene expression [4]. In recent years, a number of evidence have shown that regulation of HMTs can affect various biological characteristics of tumors [7, 8].

Cancer treatment is still challenging worldwide. Although new therapeutic drugs are being developed, such as tyrosine kinase inhibitors (TKIs), immune checkpoint inhibitors and epigenetic inhibitors [9], and these new drugs have achieved good therapeutic effects within a period of time, drug resistance might develop after initial use. Therefore, in addition to the continuous development of new drugs, solving the problem of drug resistance remains critical for cancer treatment.

Recently, many experimental results have proved that histone methylase inhibitors can reverse drug resistance [10]. In this review, we describe the relationship between various types of HMTs and their biological characteristics in cancers and drug resistance. We summarize some of the methyltransferase inhibitors that could reverse drug resistance. Interestingly, the mechanism of drug resistance is not only HMTs acting on histones, but also HMTs catalyzing non-histone proteins. Finally, we propose a new idea of “a double-target drug” for reversing cancer drug resistance.

## Classification and function of HMTs

### HMTs and its classification

**Fig. 1:**
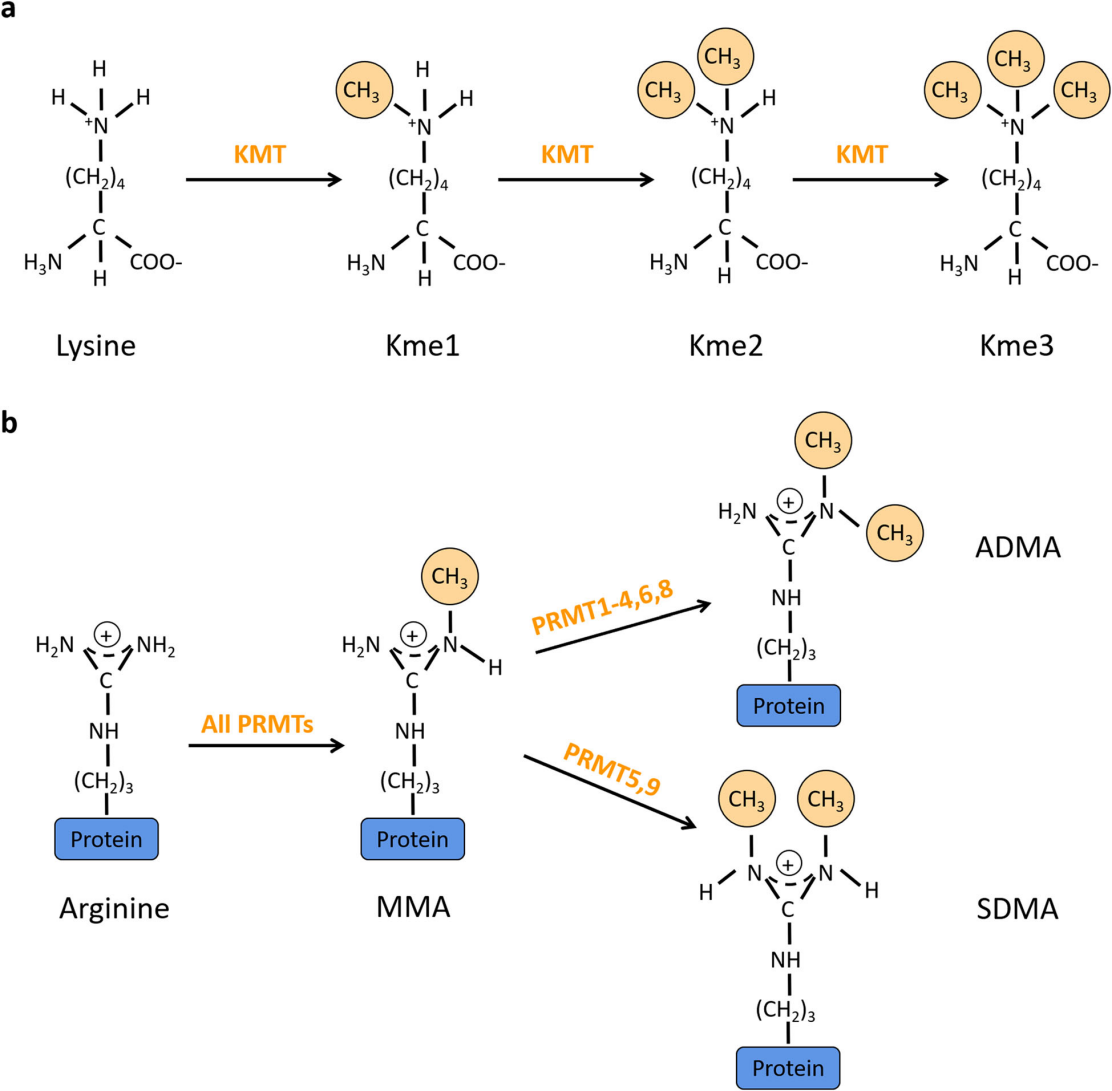
Chemical structures of methylated derivatives of histone.** a**, Chemical structures of methylated derivatives of lysine. 1–3 methyl groups on lysine side chain (shown in orange). **b**, Chemical structures of methylated derivatives of arginine. All PRMTs catalyze the production of monomethylarginine. PRMT1–4,6,8 catalyze the formation of ADMA. PRMT5, 9 catalyze the formation of SDMA

HMTs are a class of enzymes that catalyze the methyl groups of histone proteins. According to the amino acids catalyzed by this enzyme, HMTs are divided into two families: histone lysine methyltransferases (KMTs) and protein arginine methyltransferases (PRMTs). The KMTs family includes EZH2, G9a, DOT1L and SETD2; and the PRMTs family has 9 members from PRMT1–9 in mammals. In terms of catalytic sites, members of both families use S-adenosyl-L-methionine (SAM) as the methyl group donor, which catalyzes the transfer of methyl group to ε-nitrogen of the lysine side chain or arginine guanidyl nitrogen [11, 12]. Depending on the number of methyl groups added, mono-, di- and tri-methylated groups are formed in the lysine residue (Kme1, Kme2, Kme3) and mono-, and di-methyl groups are formed in the arginine residue (Rme1/MMA,Rme2). However, the arginine catalytic site has a spatial conformation that lysine does not have, thus dimethylation is divided into two types according to the spatial position of methyl groups: (1) PRMT1–4,6,8 can catalyze the addition of the second methyl group to the same nitrogen as the first methyl group to form asymmetric dimethylarginine (Rme2a or ADMA). (2) PRMT5, 9 catalyzes the second methyl group to form symmetric dimethylarginine (Rme2s or SDMA) on nitrogen different from the first methyl group [13, 14]. The above processes are shown in Fig. 1. It was remarkable that HMTs not only methylate histone, but also methylate non-histone proteins. For example, SMYD2 catalyzes monomethylation of p53 at K370 to inhibit the activities of protein [15]. PRMT1 methylates Smad6 at arginines 74 and 81 [16]. SPT5 is methylated by PRMT1 on arginines 681,696 and 698 and PRMT5 on arginines 698 [17].

Although only the primary structure has changed after methylation on lysine or arginine, methylation can lead to increased hydrophobicity and promote protein conformational change. Eventually, methylation affects downstream gene expression, therefore regulating genetic information and changing various biological functions of organisms.

### The function of HMTs

**Fig. 2:**
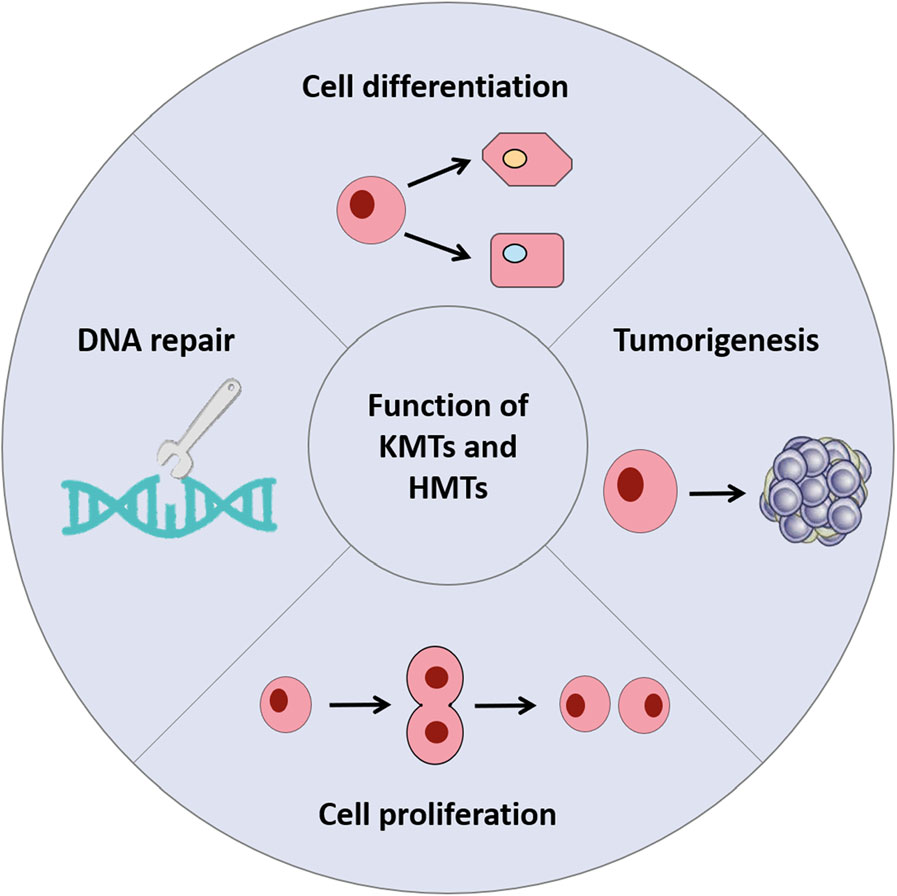
The major functions of KMTs and PRMTs. KMTs and PRMTs have a variety of functions

The HMTs, with SAM as the methyl group donor, target arginine and lysine residules on histone and excert various regulatory effects [18]. Members of the KMTs family are involved in tumorigenesis and tumor progression [19]. It has been found that KMTs participate in a variety of cellular processes, and play different roles by catalyzing lysine at different positions to regulate the expression of downstream genes. For example, *Mixed Lineage Leukemia (MLL)* is the gene which encodes histone methyltransferase and its most important downstream gene is *Homeobox* family gene. *MLL* regulates stem cell self-renewal and promots tissue or organ formation [20]. In mouse models, excision of the methyltransferase active domain (SET domain) in the *MLL* gene can cause defects in skeleton development and abnormal expression of *Homeobox*-related genes (Fig. 2) [21]. PRMTs family plays essential roles in alternative splicing, post-transcriptional regulation, RNA processing, cell proliferation, cell differentiation, apoptosis and tumorigenesis [22, 23]. For example, ADMA catalyzed by PRMT1 is involved in genes that activate cell proliferation in breast cancer, while SDMA catalyzed by PRMT5 inhibits genes of cell cycle progression [24, 25]. PRMT1 and PRMT5 can methylate the transcription elongation factor SPT5 and regulate its interaction with RNA polymerase II (Fig. 2) [17]. HMTs can also catalyze lysine or arginine sites on non-histone proteins to affect cell function and development of cancer [26]. For example, SMYD2 catalyzes p53 lysine 370 methylation to affect transcription [15]. The detail functions and mechanisms of KMTs and PRMTs can be found in pulished studies [27, 28].

## The biological characteristics of HMTs

### KMTs and tumor biological characteristics

The relationship between HMTs and tumors has been discussed in a number of publications. It was shown that HMTs have regulatory effect on tumor biological characteristics [27, 29]. The enhancer of zeste homolog 2 (EZH2) is one of the HMTs family members with catalytic activity, which can promote cancer development. EZH2 is a catalytic component of the polycomb repressive complex 2 (PRC2), which uses its HMT activity to catalyze the trimethylation of lysine at position 27 of histone H3, resulting in the suppression of downstream tumor suppressor genes such as *E-cadherin, P16 INK4α*, *P57*, and *PSP94* [30]. At present, numerous experimental results indicate that EZH2 overexpression promotes proliferation, migration and invasion of cancer cells in endometrial cancer, lung cancer, melanoma, breast cancer, bladder cancer and colorectal cancer [31–33]. EZH2 enhances cell cycle progression and activating VEGF / AKT signaling in non-small cell lung cancer (NSCLC) cells [34, 35]. In addition to overexpression of EZH2, the low expression of this family members promotes certain biological characteristics of tumors. For example, SETD2 was first described as disease-related in 1998 [36]. Alison L Clayton et al. found that SETD2 can catalyze the formation of H3K36me3 and it is the only methyltransferase for catalyzing H3K36me3 [37]. The low expression of SETD2 leads to proliferative advantage, increased colony formation and accelerated leukemia development of fusion-protein expressing cancer cells in MLL [38]. Similar reports are common in cancers such as renal cell carcinoma and pancreatic cancer [39, 40]. In addition to EZH2 and SETD2, KMTs such as G9a [41, 42], MLL [43], DOT1L [44] and NSD1 [45] are associated with tumor development and progression.

### PRMTs and tumor biological characteristics

Compared with the KMTs family, the PRMTs family members are not well investigated. However, it was found that PRMTs have different catalytic sites and catalytic activities, and they are involved in the occurrence and development of a variety of cancers. The overexpression of PRMTs can promote cancer proliferation and metastasis [46–48]. For example, PRMT1, the most active member of the PRMTs family, can catalyze methylation of the FMS-like receptor tyrosine kinase 3- internal tandem duplication protein at arginine 972/973. It can promote the proliferation of leukemia cells in acute myeloid leukaemia [49–51]. Other studies showed that overexpression of PRMT1 promotes metastasis and invasion of lung cancer through methylation of E-cadherin and Twist1 (a basic helix-loop-helix transcription factor) [52]. PRMT5 has also been reported to increase malignancy in pancreatic cancer cells via EGFR / AKT / β-catenin signaling pathway [53].

## HMTs and cancer drug resistance

### KMTs and cancer drug resistance

Cancer drug resistance is defined as therapeutic drugs loss effects on killing cancer cells and cancer patients show decrease or no effect after treatment. It was found that drug resistance is a reversible process. Although genetic mutations play roles in drug resistance, epigenetic changes may occur at a much higher frequency than genetic changes [54–56]. This section focuses on some studies on KMTs in cancer drug resistance.

#### H3K27 KMTs and drug resistance

Among the enzymes that catalyze the methylation of H3K27, EZH2 has attracted attention as a methyltransferase responsible for catalyzing H3K27me3. It is generally believed that the methylation by EZH2 makes the downstream genes difficult to express. The pro-apoptotic pathway is blocked or the anti-apoptotic pathway is enhanced in tumor cells, resulting in drug resistance [57]. However, the molecular mechanisms underlying EZH2 overexpression and its role in drug resistance remains largely unknown. It is known that the expression of EZH2 in drug-resistant cells is significantly higher than sensitive cells of ovarian cancer, multiple myeloma and other cancers. EZH2 overexpression in parental cells leads to drug resistance. Sun J et al. found that the up-regulation of EZH2 expression in drug resistant cells may be related to c-Myc in ovarian cancer. c-Myc is a transcription factor that targets genes involved in regulating cell proliferation and apoptosis. In drug-resistant cells, c-Myc inhibits miR-137 by recruiting EZH2, and miR-137 can inhibit EZH2 expression. Finally, a closed loop of c-Myc-miR-137-EZH2 is formed, which promotes the expression of EZH2 and leads to drug resistance [58]. This process causes an increase in H3K27me3 and reduces expression of cisplatin resistance-related genes including *RASSF1A, MLH1* and *CYT19* in ovarian cancer cells. Eventually leading to ovarian cancer cells resistant to cisplatin [59]. The RBPMS is a member of the RNA-binding protein family, which regulates mRNA processing and has anti-tumor effects in a variety of cancers [60–62]. The occupancy rate of EZH2 in the promoter region of this gene was significantly increased in drug-resistant cells. After inhibiting EZH2 in drug-resistant cells, the expression of RBPMS was up-regulated, which activated the G1-CDK inhibitor, p21CIP1 / WAF1, and repressed MYC and Bcl-2 transcription, leading to a decrease in drug resistance [63]. In breast cancer, Wu et al. demonstrated that EZH2 conferred tamoxifen resistance by silencing the expression of GREB1, the estrogen receptor alpha (ERα) cofactor. The mechanism might be that the use of tamoxifen can alter the activity of EZH2 and DNMTs, leading to methylation of specific CpG sites in the *GREB1* gene promoter, which reduces the expression of GREB1. However, this inhibitory effect is not through H3K27me3, but by directly governing DNA methylation with DNMTs and EZH2 acts on the CpG island of the GREB1 promoter [64]. Taken together, EZH2 induces cancer drug resistance by regulating downstream gene expression with different mechanisms. In addition to catalytic methylation, EZH2 can directly regulate downstream gene transcription.

#### H3K9 KMTs and drug resistance

KMTs modified H3K9 include G9a, SETDB1, Suv39H1 and Suv39H2. G9a is the most common KMT, which is responsible for catalyzing the formation of H3K9me1 and H3K9me2. In 2017, Liu et al. performed immunohistochemical analysis on surgically excised tumor specimens from patients with chemotherapy and found that patients with high G9a expression were less sensitive to first-line chemotherapeutic drug cisplatin than patients with low G9a expression in head and neck squamous cell carcinoma (HNSCC). Later studies also found that G9a-like protein (GLP) and EZH2 work with G9a to catalyze the methylation of histone 3 lysine-residues in HNSCC, but there was no significant correlation between GLP, EZH2 and cisplatin sensitivity. This result indicated that G9a is specific in HNSCC cisplatin resistance. In subsequent mechanistic studies, it was found that G9a promotes cisplatin resistance not by cisplatin-related transporters, but by catalyzing monomethylation of the glutamate-cysteine ligase catalytic subunit (GCLC) promoter. The methylation triggered GCLC transcriptional activation and led to upregulation of glutathione in cells. Glutathione conjugated with cisplatin, decreased DNA damage induced by cisplatin and increased cancer cell resistance to platinum drugs [65]. In lung cancer, EGFR-TKIs have good therapeutic effects, however, with the unsolvable problem of drug resistance. As early as 2007, Jeffrey et al. discovered that the cause of gefitinib resistance is MET amplification by maintaining ERBB3 (HER3) phosphorylation which leads to continuous activation of PI3K / AKT signaling pathway [66]. However, there was no answer on how to regulate HER3 to solve the problem of resistance. In 2019, Chang et al. demonstrated that G9a inhibits miR-145-5p expression after catalyzing H3K9me2 and induces HER3 expression in tumor cells by RNAseq, gene manipulation and other technologies [67]. In addition, our team demonstrated that G9a promotes H3K9me2 and reduces H3K9ac in the *PTEN* promoter region, leading to *PTEN* transcriptional repression, which leads to activation of the AKT signaling pathway and promotes EGFR-TKI resistance [68].

Suv39H1 has the catalyzing function of H3K9me3 and has been found to be related to drug resistance. Fas is a member of the death receptor superfamily, which promotes cell apoptosis and is usually expressed on the surface of tumor cells. FasL is a physiological ligand of Fas and is expressed on activated cytotoxic T lymphocytes [69, 70]. Both of them play an important role in the immune surveillance of cancer. In metastatic colon cancer cells, Paschall et al. found that Fas expression was reduced and the promoter region was rich in H3K9me3. Therefore, the cells can resist the apoptosis induced by drozitumab, the death receptor 5 agonist. With the use of Suv39H1 inhibitor Verticillin A, it was observed that the anti-apoptotic effect was reversed [71]. The above examples demonstrate that KMTs such as G9a and Suv39H1 regulate drug resistance by H3K9 methylation of key genes.

#### Other KMTs and drug resistance

In addition to KMTs modifying H3K9 and H3K27, enzymes that modify other sites have also been reported to be involved in drug resistance. For example, among the many KMTs that catalyze H3k36, SETD2 is the only enzyme in cells that catalyzes the synthesis of H3K36me3. Interestingly, it was found that *SETD2* is a tumor suppressor gene. It often causes drug resistance due to a loss of function mutation in non-solid tumors. In relapsed acute leukemia, Dong et al. found that SETD2 inactive mutant leukemia cells display down-regulated signals on S- and G2/M- checkpoint regulation and cell cycle progression. These two checkpoints mediate DNA replication and mitosis. The therapeutic mechanism of cytarabine is to withdraw cells from S phase and enter G2/M phase. Therefore, checkpoint defects caused by SETD2 inactive mutation accelerate the development of leukemia and lead to resistance to standard cytarabine-based chemotherapy [72]. SETD2 has been reported to be involved in sunitinib resistance in renal cell carcinoma. By shRNA technology, it was determined that deletion of SETD2 leads to reduce signal transduction of pro-survival MCL-1 protein, which is the downstream of the ERK and GSK3β signaling pathways. Therefore deletion of SETD2 results in sunitinib resistance, although the specific regulatory process needs further investigation [73].

It has been found that knockout of *SETD2* gene can effectively reduce cisplatin-induced ERK activation, up-regulate Bcl-xL expression, and lead to cisplatin resistance in non-small cell lung cancer [74]. DOT1L is the only KMT with a 7β chain structure in mammals, and mediates mono-, di- and trimethylation of H3K79. In studies of cisplatin resistant ovarian cancer, DOT1L is thought to regulate the development of drug resistance. The possible mechanism is that CCAAT / enhancer-binding protein β (C/EBPβ, also known as CEBPB) regulates the level of H3K79 methylation on drug resistant genes in transcription start sites by enhancing the binding capacity of DOT1L to target genes, thereby increasing tumor cells resistant to cisplatin. These downstream genes have been shown to be related to three aspects: (1) Drug transport (multidrug resistance proteins MRP1 and MRP3), (2) DNA damage repair and (3) Cell survival [75]. DOT1L has also been shown to be associated with drug resistance in human HNSCC. Lilly et al. identified that cancer stem cells are characterized with high levels of CD44V3 and aldehyde dehydrogenase-1 (ALDH1). Subsequent study found that matrix hyaluronan up-regulated the expression of DOT1L in cancer stem cells, which increased the H3K79me1 of the miR-10b promoter and caused overexpression of RhoGTPases and survival proteins, leading to cisplatin resistance in HNSCC [76]. The above examples indicate that KMTs induce drug resistance by regulating the methylation of promoter of downstream genes. It is noteworthy that inactivation of some KMTs in tumors can also lead to drug resistance [72]. Therefore, the function of KMTs in different cancers should be further explored.

### PRMTs and cancer drug resistance

The role of PRMTs family members on cancer drug resistance is less studied compared with KMTs. Most studies on PRMTs are mainly focus on PRMT1 and PRMT5.

#### PRMT1 and drug resistance

More than 75% of the catalysis by PRMTs is performed by PRMT1. Studies on PRMT1 and drug resistance are mainly reflected in different tumors resistant to cetuximab. Cetuximab is a drug targeting EGFR. In colorectal cancer, PRMT1 catalyzes the methylation of the EGFR extracellular domains R198 and R200. It enhances its binding to EGF and subsequent receptor dimer binding. EGFR methylation causes signal activation and cell proliferation, and cetuximab resistance in patients with colon cancer [77]. In HNSCC, Hsu et al. found that epithelial-mesenchymal transition induced by Snail occurrs in patients with HNSCC resistant to cetuximab. Mechanistic studies show that Snail induces the expression of lymphotoxin-β, a TNF superfamily protein that activates the expression of PRMT1. Lymphotoxin-β can interact with PRMT1 after it causes methylation of EGFR extracellular domains R198 and R200. Eventually, improve the ligand binding capacity of EGFR and promote the dimer formation, leading to drug resistance [78]. In addition cetuximab resistance, increased PRMT1 modification was observed in cisplatin-resistant ovarian cancer cells. The PRMT1 modification mainly involved chromatin and related proteins in causing genotoxic stress. It was confirmed that PRMT1 catalyzes H4R3, thereby participating in the activation of genes related to senescence-associated secretory phenotype (SASP) by regulating the NF-kB pathway. This gene protects cells from DNA damage, thereby increasing tumor cell resistance to cisplatin [79]. In conclusion, compared with KMTs, PRMT1 promotes drug resistance through methylation of the promoter region of downstream genes. In addition, PRMT1 regulates drug resistance by catalyzing the arginine residue to regulate the function of proteins.

#### PRMT5 and drug resistance

PRMT5 is another prominent member of PRMTs that catalyzes SDMA and results in suppressing downstream gene transcription, which induces stemness of breast cancer cells [80]. A study shows that FOXP1 is a key factor in PRMT5-induced breast cancer stem cells and PRMT5 can be recruited to the FOXP1 promoter to facilitates recruitment of H3R2me2s, SET1 and H3K4me3 [81]. FOXP1 is a member of the forkhead box transcription factor family and is associated with cancer cell stemness [82]. FOXP1 can also promote tumor proliferation and migration [83]. In glioblastoma (GBM), PRMT5 mediats symmetric dimethylation of heterogeneous nuclear ribonucleoprotein 1 (hnRNP A1) at arginine residues 218 and 225. These methylations are sufficient to promote hnRNP A1 binding to cyclin D1 and c-Myc IRESs, and result in resistance to mTOR inhibitors [84]. In addition, there have been reports that PRMT5 and H4R3me2s bind to the promoter region of the *hepatocyte nuclear factor 4α* (*HNF4α*) gene in liver cancer cells. The binding leads to a decrease in HNF4α transcription. HNF4α is a transcription factor responsible for liver cell transformation. It usually inhibits the occurrence of EMT in liver cells and the occurrence of liver cancer cell stemness. The emergence of stem cells promotes drug resistance in liver cancer cells after PRMT5 suppressed the expression of HNF4α [85]. The above situation is consistent with the general recognition that PRMT5 overexpression leads to drug resistance. However, a checkpoint regulatory protein Rad9, was reported to be catalyzed by PRMT5 to produce methylation at Arg172, Arg174 and Arg175. And this methylated Rad9 protein is DNA damage dependent. Studies show that knocking out PRMT5 reduces methylation and defective S / M checkpoints. Eventually cells will not sensitive to the DNA synthesis inhibitor hydroxyurea [86]. Therefore, the same PRMT may have paradoxical effects on drug resistance in different tumors, although the mechanism of action might be similar.

#### Other PRMTs and drug resistance

**Fig. 3:**
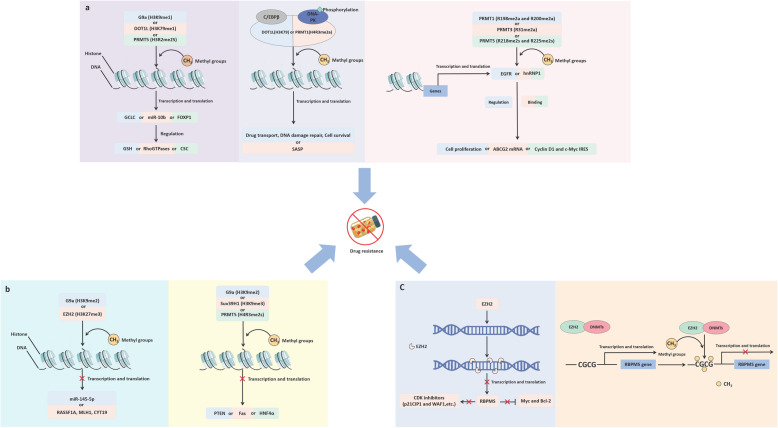
The mechanism of HMT induced drug resistance. **a**, KMTs and PRMTs catalyze the methylation of gene promoters (3a left) or transcription start sites (3b middle) alone or with other molecules (3b right), which activates the transcription of downstream drug resistance-related genes, resulting in drug resistance. PRMTs promote the binding of proteins to other molecules by catalyzing protein arginine residue, leading to drug resistance. **b**, KMTs and PRMTs catalyze methylation of promoter (3b right) or “unknown” regions that are not a promoter (3b left). This leads to suppression of transcription of tumor suppressor genes or genes that induce tumor stem cell formation, leading to drug resistance. **c**, EZH2 directly acts on the promoter region by itself (3c left) or with DNMTs (3c right) to methylate CpG islands in the promoter, resulting in transcriptional repression of downstream genes

In gemcitabine-resistant pancreatic cancer cells, Chuan et al. found that PRMT3 can catalyze ADMA at the 31-arginine of its interacting protein hnRNP A1. And the methylated hnRNP A1 can bind to a member of the ATP-binding cassette subfamily G member 2 (ABCG2) mRNA, which plays a key role in drug resistance. This binding increases ABCG2 mRNA stability. ABCG2 expression is increased leading cancer cells resistant to many drugs including gemcitabine [87]. The mechanism of HMTs leading to drug resistance are shown in Fig. 3.

## Regulating HMTs on drug resistance in tumors

### KMT inhibitors reverse cancer drug resistance

#### Targeting H3K27 KMTs reverse drug resistance

The relationship between HMTs and cancer drug resistance has gradually been valued, therefore, the idea of reversing drug resistance by regulating HMTs has emerged. This section focuses on the reversal effect of KMT inhibitors on drug resistance in recent years.

EZH2 has attracted the most attention as a KMT, and its inhibitors in reversing drug resistance in various cancers have been reported. First, (1) Small molecular drugs. Receptor tyrosine kinase inhibitors, such as sunitinib, have been accepted as a standard treatment among patients with metastatic renal cell carcinoma. However, drug ressitance develop with long time use of the drug [88]. Studies from tumor models and clinical trials found that the drug resistance can be reversed to some extent by increasing the drug dose. Therefore, it is assumed that the resistance of sunitinib in this disease is regulated by epigenetics [89]. Adelaiye-Ogala et al. discovered that EZH2 overexpression triggers overall phosphorylation of kinases in serine and tyrosine residues and causes drug resistance. The inhibition of EZH2 by its inhibitor EPZ011989 can reduce phosphorylation and activate tumor suppressors to reverse drug resistance [90]. Second, (2) Classic chemotherapeutic drugs. Compared with sunitinib, classic chemotherapeutic drugs, such as cisplatin, 5-FU and others have more severe drug resistance. Ovarian cancer is one of the diseases known as the “female killer”. The most commonly used drug in ovarian cancer is cisplatin and its derivatives, but more than half of patients develop drug resistance in different stages of treatment. The mechanism of drug resistance is believed to be the increase of cellular drug efflux, resulting in the decrease of intracellular platinum [91]. In the follow-up study, it was found that EZH2 increases the degradation of copper transporter 1, which is a high-affinity copper influx transporter closely related to platinum resistance. Copper transporter 1 decreases the accumulated platinum content inside cells, which leads to drug resistance. However, after adding the EZH2 inhibitor GSK126 for 12 h, the copper transporter 1 content was significantly higher than that of the untreated cells [92]. Drug resistance occurs in small cell lung cancer (SCLC), with the first-line treatment of platinum drugs and etoposide. However, the latest research by Qiu et al. suggested that the resistance mechanism may be related to cyclin-dependent kinase inhibitor 1C (CDKN1C, P57Kip2) regulated by Chromodomain Y-like. This regulation is carried out by recruiting EZH2 to catalyze H3K27me3 in the CDKN1C promoter region, which causes transcriptional repression. Therefore, the EZH2 inhibitor GSK126 can increase the expression of CDKN1C and increase drug sensitivity [93]. Finally, (3) Immunotherapeutic drugs. The use of immunotherapeutic drugs is a new treatment modelity in recent years. They are very effective at the beginning of treatment, but drug resistance occurred after a period of treatment. In the immunotherapy using anti-CTLA-4 or IL-2 in mice, the production of tumor necrosis factor-a (TNF-a) in tumor cells and the accumulation of T cells led to an increase in EZH2 expression in melanoma cells. This process leads to drug resistance due to inhibition of its own immunogenicity and antigen presentation. Treatment with the EZH2 inhibitor GSK503 reduces the amount of H3K27me3. Also, GSK503 down-regulates PD-L1 expression and successfully reverses drug resistance in melanoma cells [94].

#### Targeting H3K9 KMTs reverse drug resistance

KMT catalyzing the methylation of H3K9. It has been reported that the use of G9a inhibitors can reverse drug resistance. Our team has demonstrated that G9a is associated with EGFR-TKI resistance in NSCLC. We found that the use of G9a inhibitor UNC0638 can significantly inhibit the growth of drug-resistant cancer cells and induce cell apoptosis. By evaluating the expression of key molecules in certain pathways in drug-resistant cells and parental cells, we found that the drug-resistance is associated with activation of AKT signaling pathway. Further study indicated that G9a activates the AKT signaling pathway by increasing the level of H3K9me2 in the *PTEN* promoter region and reducing acetylation, which leads to drug resistance [68]. In terms of classic chemotherapeutic drugs, we found that the use of G9a inhibitor UNC0638 in drug-resistant cells can significantly reduce GCLC expression in cisplatin resistant HNSCC. Using the luciferase reporter gene detection analysis, it was found that inhibition of G9a activity directly inhibits *GCLC* transcriptional activation. The effect was achieved by regulating the level of H3K9me1 at the *GCLC* transcription initiation site, and GCLC eventually caused changes in intracellular Glutathione expression to reverse drug resistance [65]. Ciechomska et al. found that the application of G9a specific inhibitor BIX-01294 causes GBM cells to resensitize to temozolomide, but its mechanism remains to be explored [95]. In a gemcitabine-resistant cervical cancer model, Candelaria et al. observed changes in the expression of two genes related to gemcitabine resistance. The first one is *human Equilibrative Nucleoside Transporter 1 (hENT1)*, which has been shown to mediate intracellular uptake of gemcitabine. The second one is *deoxyeytidine kinase*, a protein associated with gemcitabine sensitivity. It was found that the H3K9me2 level in the promoter of the two genes rises after gemcitabine treatment and suggested that G9a expression in drug-resistant cells is higher than that in sensitive cells. Then, by detecting mRNA and protein levels, it was found that hydralazine can reduce the expression level of G9a and H3K9me2 in the promoter of two genes in drug-resistant cells, thereby reversing gemcitabine resistance [96]. In terms of targeted drugs, hepatocellular carcinoma (HCC) has been reported to be resistant to tumor necrosis factor-related apoptosis-inducing ligand (TRAIL), which is a cancer-selective, cell-death-inducing agent with little toxicity to normal cells. The G9a inhibitor BIX-01294 can reverse drug resistance by down-regulating the expression of Survivin, which is a member of the inhibitor of apoptosis protein family. However, how G9a regulates the change of Survivin and the relationship between them needs further studied [97].

#### Targeting other KMTs reverse drug resistance

The understanding of KMT catalyzing sites is limited, and there is few reports on the research progress of their relationship with cancer drug resistance or the development of inhibitors. This section summarizes the existing reports. First, SETD2 was found to catalyze the methylation of H3K36 and have an anti-cancer effect. Unlike other KMTs that catalyze methylation to cause cancer development and cancer drug resistance, methylation induced by SETD2 can actually inhibit cancer growth and reverse drug resistance. In leukemias, SETD2 usually undergoes loss of function mutations and deletions [27]. It was found that tumor cells with these changes significantly reduced their DNA damage response after exposure to cytotoxic chemotherapeutic drugs such as cytarabine and etoposide [98, 99]. The results are reduced cell apoptosis and decreased sensitivity to chemotherapy. The mechanism of this phenomenon was due to the loss of H3K36me3 in exons of proteins related to homologous recombination and mismatch repair after SETD2 deletion, such as lens epithelium-derived growth factor (LEDGF) and MutS Homolog 6 (MSH6) [100, 101]. In terms of treatment, the inactive mutation or loss of *SETD2* is difficult to recover. JIB-04 can reverse drug resistance by inhibiting KDM4A (H3K9 and H3K36 demethylase) to increase the level of H3K36me3 [99]. Furthermore, DOT1L is the only enzyme that can catalyze H3K79, and reverse drug resistance by regulating the expression of ERα. The expression and regulation of ERα protein have been identified as the main reason for breast cancer cells tolerant hormonal drugs, but how to block the ERα pathway is still a difficult problem. Nassa et al. found that the use of the DOT1L inhibitor EPZ004777 on estrogen-resistant cells and ectopic transplantation animals can inhibit the binding of DOT1L in the C-terminal of ERα. At the same time, it was found that H3K79 mono-methylation, di-methylation and tri-methylation were reduced in the transcriptional regions of related genes in drug-resistant cells. Eventually this drug reversed the resistance to homonal drugs in breast cancer [102]. KMT, which catalyzes H3K4, has also been reported to reverse drug resistance. Lu et al. reported that the mRNA and protein levels of MLL1, and the transcription level of PD-L1 are higher in pancreatic cancer cells than that in normal pancreatic cells. Subsequent research proved that MLL1 catalyzed the formation of H3K4me3 after enrichment in the *CD274* promoter, and it activates the expression of PD-L1 in tumor cells and leads to drug resistance. However, after using the MLL1 inhibitor verticillin A, it was observed that the level of H3K4me3 in the *CD274* promoter region is decreased and the expression of PD-L1 in cells is decreased, thereby enhancing the efficacy of anti-PD-L1 immunotherapy [103].

### PRMT inhibitors reverse cancer drug resistance

While KMT inhibitors were found to be able to reverse drug resistance, the question of whether PRMT inhibitors have similar functions was quickly answered. Cyclin-dependent kinase 4/6 (CDK4/6) inhibitors are well-recognized drugs in the treatment of estrogen receptor-positive breast cancer. However, drug resistance to CDK4/6 inhibitors occurred in melanoma treatment. Analysis of drug-resistant cells indicates that PRMT5 activity is critical to the sensitivity of CDK4 / 6 inhibitors. AbuHammad et al. found that PRMT5 inhibitor GSK3326595 reverses drug resistance of CDK4 / 6 inhibitor palbociclib in melanoma cells. The mechanism may be due to the down regulation of PRMT5, which leads to inhibiting p53 negative regulator MDM4 expression and activation of p53. Activation of p53 inhibits the expression of CDK4 and the kinase of CDK4 / 6, leading to reverse drug resistance [104]. IRES has been identified as the primary form of resistance to mTOR inhibitors. EPZ015666, the other inhibitor of PRMT5, can inhibit the PRMT5 mediated SDMA methylation of hnRNP A1 in gliomas. This can inhibit the activation of related proteins mediated by IRES and reverse the resistance of glioma cells to mTOR inhibitors [84]. The mechanisms that regulate KMTs and PRMTs to reverse drug resistance are summarized in Table 1.

**Table 1:** Effect of regulating HMTs on drug resistance in tumors

Enzyme	Cancer type	Drug	Inhibitor	Mechanism (Reference)
EZH2	Clear cell renal cell carcinoma	Sunitinib	EPZ011989	Reduced overall phosphorylation of kinases and increased activation of tumor suppressors [90].
	Ovarian cancer	Cisplatin	GSK126	Increased copper transporter 1 & platinum accumulation [91, 92].
	SCLC	Etoposide	GSK126	Reduced H3K27me3 in *CDKN1C* promoter, leading to increased CDKN1C expression [93].
	Melanoma	Immune checkpoint inhibitors	GSK503	Decreased H3K27me3 leads to up-regulation of antigen and down-regulation of PD-L1 [94].
G9a	NSCLC	EGFR-TKI	UNC0638	Regulation of PTEN / AKT pathway is inhibited [68].
	HNSCC	Cisplatin	UNC0638	H3K9me1 reduction in *GCLC* promoter causes an increase in Glutathione expression [65].
	GBM	Temozolomide	BIX-01294	Unknown [95].
	Cervical cancer	Gemcitabine	Hydralazine	H3K9me2 decreases in *hENT1* and *deoxyeytidine kinase* promoters [96].
	Hepatocellular Carcinoma	TRAIL	BIX-01294	Downregulate Survivin [97].
SETD2	Acute leukemia	Cytarabine, Etoposide	JIB-04	Reduced H3K36me3 in exons of LEDGF and MSH6 related proteins [99–101].
DOT1L	Breast cancer	Antiestrogen	EPZ004777	Blocked ERα expression [102].
MLL1	Pancreatic cancer	PD-L1 inhibitor	Verticillin A	Reduced H3K4me3 in the *CD274* promoter and PD-L1 expression [103].
PRMT5	Breast cancer	CDK4/6 inhibitor (palbociclib)	GSK3326595	Reduced MDM4 expression leads to p53 activation and the inhibition of CDK2 [104].
	GBM	mTOR inhibitors	EPZ015666	Inhibited SDMA methylation in hnRNP A1 and IRES-mediated activation of related proteins [84].

### HMTs and other molecular targets synergistically inhibit cancer drug resistance

HMTs alone could be able to reverse drug resistance. However, there have been reports support “a double-target drug” concept porposed by our team. “A double-target drug” refered to the coordination of HMTs with other molecular targets to regulate tumor drug resistance. For example, in chemotherapy-resistant GBM, De La Rosa et al. found that 3-deazaneplanocin A (an EZH2 inhibitor) combined with panobinostat (an HDAC inhibitor) reduces tumor resistance and increases cell apoptosis. Therefore, the combination of 3-deazaneplanocin A and panobinostat is considered as a great strategy for treating GBM [105]. Thalidomide and its derivatives, lenalidomide and pomalidomide (also known as IMiDs) could be an effective way to treat multiple myeloma. The combination of 5-azacytidine (an DNMT1 inhibitor) and EPZ-6438 (an EZH2 inhibitor) can almost entirely reverse the chromatin accessibility to the initial state and restore drug sensitivity [106].

## Conclusions and future directions

**Fig. 4:**
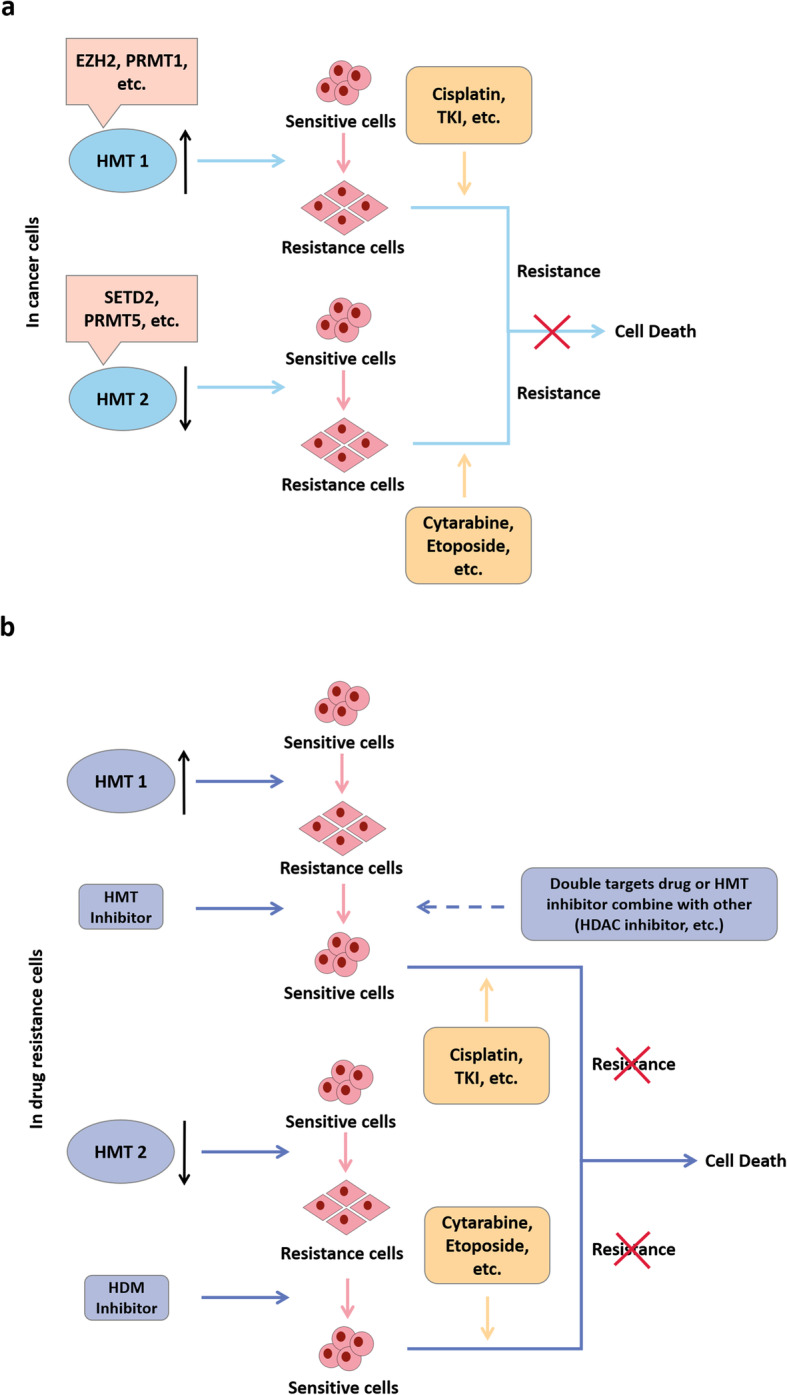
Targeting HMTs reverses drug resistance. **a**, In cancer cells, HMT 1 up-regulation or HMT 2 down-regulation leads to drug resistance. **b**, Drug resistance caused by HMT 1 can be reversed by corresponding inhibitors. A double-target drug or HMT inhibitor combine with others (HDAC inhibitor, et al.) reverse resistance. Drug resistance caused by HMT 2 can be reversed by HDM inhibitors

Regulation of HMTs family members is reversible and is involved in tumorigenesis and drug resistance. A number of studies have suggested that the mechanism of cancer drug resistance is complex and related to multiple genes. HMTs regulate genes associated with drug resistance and may work synergistically with other drugs to reverse drug resistance. Therefore, targeting HMTs may be an important breakthrough to overcome drug resistance. HMTs can be divided into HMT 1 and HMT 2. HMT 1 represents KMTs or PRMTs that are up-regulated in tumors and cause drug resistance. HMT 2 represents KMTs or PRMTs that are down-regulated in tumors and cause drug resistance. The treatment strategies for HMT 1 and HMT 2 are as follows: (1) HMT 1. Inhibitors are used to suppress their effects. If the inhibitor and other molecular target drugs have a characteristic of synergistic reversal of drug resistance, they can be used simultaneously. (2) HMT 2. Demethylase inhibitors can be used to reverse drug resistance if the drug resistance mechanism is related to modification of methylation (Fig. 4). It is noteworthy that different catalytic sites can cause different regulatory effects, and they are often unable to show consistent functions due to heterogenety of cancers. Therefore, the mechanism of drug resistance caused by different HMTs in different tumors needs further exploration.

Based on the fact that HMTs and HDAC synergistically reverse drug resistance and the role of HMTs in tumor regulation, we propose a idea that there may be interactions between HMTs, or between HMTs and other molecular targets, it is important to clarify the interaction between those factors and design “a double-target drug” for the successful treatment of cancers.

## Data Availability

Not applicable.

## Abbreviations

HMT: Histone methyltransferase
TKI: Tyrosine kinase inhibitor
KMT: Lysine methyltransferase
PRMT: Protein arginine methyltransferase
SAM: S-adenosyl-L-methionine
Kme1: Lysine-monomethylation
Kme2: Lysine-dimethylation
Kme3: Lysine-trimethylation
Rme1/MMA: N-monomethylarginine
ADMA: Asymmetric dimethylarginine
SDMA: Symmetric dimethylarginine
MLL: Mixed lineage leukemia
EZH2: Enhancer of zeste homolog 2
PRC2: Polycomb repressive complex 2
NSCLC: Non-small cell lung cancer
ERα: Estrogen receptor alpha
HNSCC: Head and neck squamous cell carcinoma
GLP: G9a-like protein
GCLC: Glutamate-cysteine ligase catalytic subunit
C/EBPβ: CCAAT / enhancer-binding protein β
ALDH1: Aldehyde dehydrogenase-1
SASP: Senescence-associated secretory phenotype
GBM: Glioblastoma
hnRNP A1: Heterogeneous nuclear ribonucleoprotein 1
HNF4α: Hepatocyte nuclear factor 4α
ABCG2: ATP-binding cassette subfamily G member 2
SCLC: Small cell lung cancer
CDKN1C: Cyclin-dependent kinase inhibitor 1C
TNF-a: Tumor necrosis factor-a
hENT1: Human equilibrative nucleoside transporter 1
HCC: Hepatocellular carcinoma
TRAIL: Tumor necrosis factor-related apoptosis-inducing ligand
LEDGF: Lens epithelium-derived growth factor
MSH6: MutS homolog 6
CDK4/6: Cyclin-dependent kinase 4/6
